# Cost-effectiveness analysis of tislelizumab vs. camrelizumab for the treatment of second-line locally advanced or metastatic esophageal squamous cell carcinoma

**DOI:** 10.1186/s12913-024-11142-5

**Published:** 2024-05-29

**Authors:** Pingyu Chen, Chang Fu, Lin Shen, Zhengyang Fei, Mengjie Luo, Yanqiu Chen, Hongchao Li

**Affiliations:** 1 Department of Health Economics, China Pharmaceutical University, Nanjing, China; 2 Center for Pharmacoeconomics and Outcomes Research, China Pharmaceutical University, Nanjing, China; 3 Department of Gastroenterology, Peking University Cancer Hospital, Beijing, China

**Keywords:** Economic evaluation, ESCC, PD-1 inhibitors, Tislelizumab, Camrelizumab

## Abstract

**Background:**

Esophageal carcinoma is a type of cancer that occurs in the esophagus. For patients with locally advanced or metastatic esophageal squamous cell carcinoma who have either experienced disease progression following first-line standard chemotherapy or are intolerant to it, the prognosis is typically poor. Additionally, these patients often bear a substantial economic burden during the course of their treatment. Tislelizumab is a selective PD-1 inhibitor with efficacy proven in locally advanced or metastatic esophageal squamous cell carcinoma. The study aims to evaluate the cost-effectiveness of tislelizumab versus camrelizumab as the second-line treatment in locally advanced or metastatic esophageal squamous cell carcinoma (ESCC) patients in China.

**Methods:**

From the perspective of China’s healthcare system, the partitioned survival model with three health states was established in a 3-week cycle and a lifetime horizon. Anchored matching adjusted indirect comparison was used for survival analyses based on individual patient data from RATIONALE 302 trial and the published ESCORT study due to the lack of head-to-head clinical trials. Only direct medical costs were included. Costs and utility values were derived from local charges, the published literature, and related databases. Sensitivity analyses and a scenario analysis were also performed to verify the robustness of the model results.

**Results:**

Compared with camrelizumab monotherapy, tislelizumab monotherapy incurred a lower lifetime cost ($8,346 vs. $8,851) and yielded higher quality-adjusted life-years (QALYs) (0.87 vs. 0.63), which resulted in an incremental cost-effectiveness ratio (ICER) of -$2,051/QALY. Tislelizumab monotherapy is a dominant option over camrelizumab monotherapy in China. The three primary parameters upon which this result was most sensitive were the unit cost of camrelizumab, the unit cost of tislelizumab, and the duration of reactive cutaneous capillary endothelial proliferation (RCCEP). According to the probabilistic sensitivity analysis (PSA), tislelizumab monotherapy was 100% cost-effective when the WTP was 1–3 times GDP per capita in China($11,207/QALY∼$33,621/QALY). Scenario analysis showed that the result was consistent.

**Conclusion:**

Tislelizumab monotherapy is a dominant option compared with camrelizumab monotherapy as the second-line treatment for locally advanced or metastatic ESCC in China.

**Supplementary Information:**

The online version contains supplementary material available at 10.1186/s12913-024-11142-5.

## Introduction

Esophageal cancer is the seventh most common malignancy worldwide [1]. According to GLOBOCAN 2020, there were approximately 604,100 newly diagnosed esophageal cancer cases globally, accounting for 3.1% of all new cases of cancer in 2022 [1]. The incidence of esophageal cancer has significant geographical and economic variations, with obviously higher rates in Asia than in other regions [2]. In China, the incidence of esophageal cancer has been decreasing in recent years [3]. The number of new cases in 2020 was 324,422, accounting for 7.1% of all new tumor cases. Esophageal adenocarcinoma and squamous cell carcinoma are the two main histological subtypes of esophageal cancer, of which esophageal squamous cell carcinoma (ESCC) is the predominant subtype worldwide [4]. ESCC accounts for approximately 88.81% of all esophageal cancers in China [5]. Although the prognosis of patients with esophageal cancer has improved since the development of medical and health technology in recent years, ESCC still has a poor 5-year survival rate of less than 20% due to its late diagnosis stage and poor prognosis [6], which bring a heavy disease burden to society.

According to the *Guidelines of Chinese Society of Clinical Oncology (CSCO) for Esophageal Cancer* [7], chemotherapy is commonly used as the first-line treatment for patients with advanced or metastatic ESCC, such as fluorouracil plus platinum-based chemotherapy. For locally advanced or metastatic ESCC patients who have failed after first-line chemotherapy treatment, immunotherapy is an option as second-line treatment. Tislelizumab, an innovative humanized lgG4 anti-programmed death receptor 1 (PD-1) monoclonal antibody developed in China, is recommended with Class 1A evidence for the second-line treatment of locally advanced or metastatic ESCC in the guideline [7]. It was approved by National Medical Products Administration (NMPA) as the second-line treatment of patients with locally advanced or metastatic ESCC in April 2022 based on the results of clinical trial RATIONALE 302. RATIONALE 302 [8] is a randomized, open-label, multicenter, global phase III clinical trial comparing the efficacy and safety between tislelizumab monotherapy and investigator-chosen chemotherapy (ICC) as second-line treatment in locally advanced or metastatic ESCC. The results of the study indicated that tislelizumab monotherapy significantly prolonged patient overall survival (OS) compared to chemotherapy regimens (8.6 months vs. 6.3 months, *P* = 0.0001) in the overall population. In the Asian subgroup, tislelizumab monotherapy also brought significantly higher overall survival benefits than chemotherapy (8.5 months vs. 6.3 months).

In addition, camrelizumab is another PD-1 inhibitor recommended by the *Guidelines of CSCO for Esophageal Cancer* as Class 1A evidence for the second-line treatment of advanced ESCC [7]. ESCORT [9], a multicenter, randomized, open-label, phase III clinical trial, compared the efficacy and safety of camrelizumab monotherapy with investigator’s choice of chemotherapy for the treatment of locally advanced or metastatic ESCC patients in China. The results of the study showed that the median OS of camrelizumab monotherapy was 8.3 months, compared with 6.2 months in patients treated with chemotherapy. Currently, camrelizumab monotherapy and tislelizumab monotherapy as second-line treatment for locally advanced or metastatic ESCC were included in the *National Medical Insurance Drug List* (NMIDL) in China, which greatly improved patient accessibility and affordability since a great amount of drug costs are covered by the social medical insurance plans. Camrelizumab and tislelizumab were proven to be cost-effective as it is listed on NMIDL for second-line treatment for locally advanced or metastatic ESCC. However, the cost-effectiveness of tislelizumab monotherapy as second-line treatment for locally advanced or metastatic ESCC in comparison with camrelizumab monotherapy in China has not been evaluated due to the lack of head-to-head clinical trials of tislelizumab monotherapy versus camrelizumab monotherapy and it cannot be determined that camrelizumab, is the most cost-effective.

The aim of this study was to evaluate the cost-effectiveness of tislelizumab monotherapy compared with camrelizumab monotherapy as the second-line treatment of locally advanced or metastatic ESCC from the perspective of China’s healthcare system.

## Methods

### Model structure

Both the Markov model and partitioned survival model (PSM) are the most frequently used approach in the cancer health economic evaluation. A published systematic review [10] indicated that although the Markov model and PSM yielded similar results under the same model structure and assumptions, the PSM was more recommended and was easier to construct when individual patient data (IPD) were available. Furthermore, the PSM is the most common model employed in evaluating the cost-effectiveness of cancer treatment according to a review of the *National Institute for Health and Care Excellence (NICE) appraisals* [11].

**Fig. 1:**
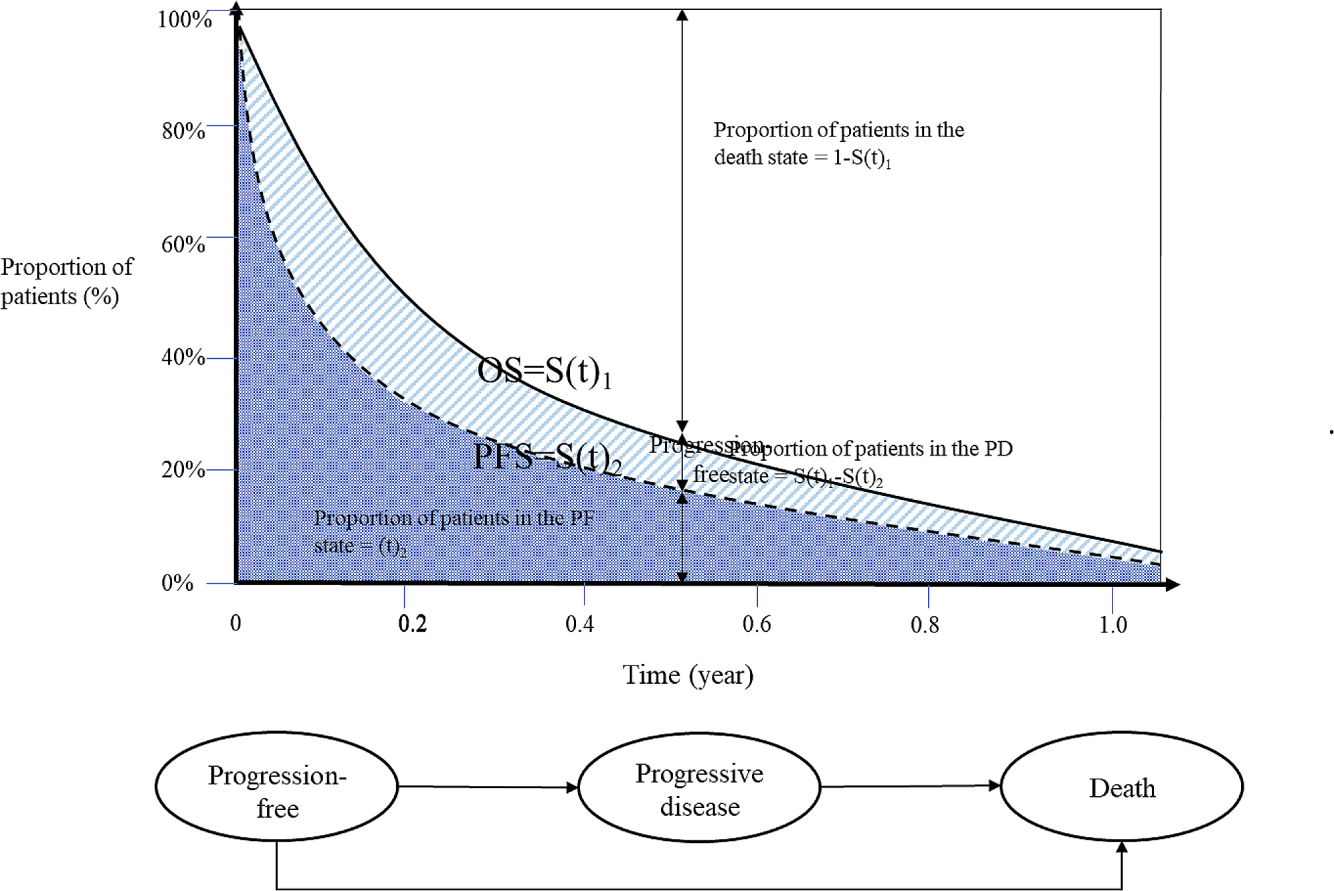
The trapezoidal method and partitioned survival model structure

Therefore, a PSM was developed in Microsoft Excel to evaluate long-term health outcomes and costs for tislelizumab monotherapy versus camrelizumab monotherapy as the second-line treatment of locally advanced or metastatic ESCC. Three mutually exclusive states were included in the model, namely progression-free (PF), progressive disease (PD), and death. All patients were assumed to be in the PF state initially, and either stay in the same health state or move to another health state in the next cycle. The period of patients in each health state over time is determined by the area under the curve (AUC) of a set of mutually exclusive survival curves which commonly are overall survival (OS) curves and progression-free survival (PFS) curves [12]. The trapezoidal method was applied to calculate the AUC, as illustrated in Fig. 1. According to the partitioned survival model, the number of individuals in the queue in the PF state and the OS state is determined by the PFS and OS survival curves. The number of individuals in the PD state is calculated by subtracting the number of individuals in the PFS state from the number of individuals in the OS state. The number of individuals in the dead state is calculated by subtracting the total number of individuals from the number of individuals in the OS state.

The quality-adjusted life-years (QALYs), life-years (LYs), and costs were all measured in this model. The cycle length of the model was 3 weeks, which was aligned with the medication administration cycle in RATIONALE 302, and the time horizon was lifetime (defined by 99% of the patient cohort dead). Only direct medical costs were taken into consideration from China’s healthcare system perspective. Patients in the PF state were assumed to have treatment-related costs, including drug costs, follow-up costs, disease management costs, and costs of adverse events (AE) management. Within the PD state, costs associated with disease management, follow-up visit, and subsequent treatment would occur. If patients died, the cost of end-of-life care was calculated. All costs and health outcomes were calculated based on the 2021–2022 prices and discounted at 5% annually according to the *China Guidelines for Pharmacoeconomic Evaluations 2020* [13]. All costs have been converted into US dollars, and the exchange rate was 1 US dollar = 7.23 Chinese RMB Yuan (US Dollar to Chinese Yuan Renminbi conversion – Last updated 20 October 2022, 06:19 UTC). In addition, the threshold of willingness to pay (WTP) for a QALY was assumed to be 1–3 times GDP per capita of China ($11,207–$33,621 per QALY gained in 2021).

### Patient population

Based on the trial eligibility criteria of ESCORT and RATIONALE 302, the target patient population of the model was patients with a histologic or cytologic diagnosis of locally advanced or metastatic ESCC who had progressed on or were intolerant to first-line chemotherapy. This study focuses on a patient population consistent with the enrollment criteria and characteristics of participants in the RATIONALE 302 and ESCORT trials. For the sake of comparability between the two trials and to ensure consistency, the analysis concentrates on the Chinese subpopulation within the RATIONALE 302 trial, including Taiwan, while excluding patients over the age of 75 and those with brain metastases. The choice to restrict the analysis to this subpopulation was made to align the baseline characteristics as closely as possible and reduce bias when indirectly comparing tislelizumab monotherapy from RATIONALE 302 with camrelizumab monotherapy from the ESCORT trial. Patients in the intervention group received 200 mg tislelizumab on day 1 of each 21-day. Camrelizumab 200 mg was administered intravenously over 30 min on day 1 of each 14-day cycle for patients in the control group. All patients received tislelizumab or camrelizumab monotherapy until disease progression, unacceptably toxic adverse reactions occurred or withdrew for other reasons.

### Clinical input

Clinical data on the efficacy and safety of tislelizumab and camrelizumab were derived from RATIONALE 302 trial and the published clinical trial ESCORT, respectively [9]. Since there is no head-to-head clinical trial of tislelizumab monotherapy versus camrelizumab monotherapy, the anchored matching adjusted indirect comparison (MAIC) method was adopted in the model to adjust the baseline of patient characteristics. In the adjustment process, key baseline demographic and disease characteristics factors, namely age, gender, histological grade, disease metastasis, lymphatic metastasis, PD-L1 expression level, and Eastern Cooperative Oncology Group (ECOG) score, prior therapies (PT) surgery, PT. radiotherapy, PT. platinum-based chemotherapy, which were reported in ESCORT, were included. The results of baseline patient characteristics and clinical efficacy data before and after adjustment are shown in Tables 1 and 2.

**Table 1:** Baseline characteristics before and after adjustment

	Before adjustment			After adjustment	
Adjustment factor	Camrelizumab group [1](*N = 448)*	Tislelizumab group (*N = 495)*	*P* value unweighted	Tislelizumab group (ESS = 137)	*P* value weighted
Age ≤ 60	50%	48%	0.66	50%	1
Male	89%	90%	0.72	89%	1
Histological grade 3(Poorly differentiated)	29%	18%	0	29%	1
Disease metastasis	84%	97%	0	84%	0.91
Lymphatic metastasis	85%	76%	0	85%	1
High PD-L1 expression level (vCPS ≥ 10%)	43%	29%	0	43%	1
ECOG PS = 1	80%	83%	0.29	80%	1
PT. Surgery	49%	45%	0.25	49%	1
PT. Radiotherapy	66%	65%	0.8	66%	1
PT. Platinum-based chemotherapy	95%	98%	0.06	95%	1

Abbreviations: ECOG = Eastern Cooperative Oncology Group; PS = performance status; ESS = effective sample size; P.T. = Prior therapies

**Table 2:** Results of progression-free survival and overall survival after MAIC

Adjustment results	HR-PFS	HR-OS
**Tislelizumab monotherapy**		
Before MAIC	0.85 (0.65–1.10)	0.74 (0.58–0.95)
After MAIC	0.78 (0.55–1.11)	0.68 (0.49–0.94)
**Camrelizumab monotherapy**	0.69 (0.56–0.86)	0.71 (0.57–0.87)

HR: hazard ratio; PFS: progression-free survival; OS: overall survival; MAIC: matching adjusted indirect comparison

Six parametric distributions, including the exponential, Weibull, Gompertz, log-normal, log-logistic, and gamma distributions, were used to extrapolate the survival curves to capture survival outcomes in lifetime horizon. Since the PFS curves of tislelizumab before and after MAIC adjustment relative to the chemotherapy group and the PFS curves of camrelizumab relative to the chemotherapy group did not meet the PH assumption (Supplementary Figures S1-S2), the MAIC-adjusted HR values were not used to adjust the efficacy. The standard parameters were fitted separately for the camrelizumab monotherapy in ESCORT and the adjusted tislelizumab monotherapy in RATIONALE 302.

**Fig. 2:**
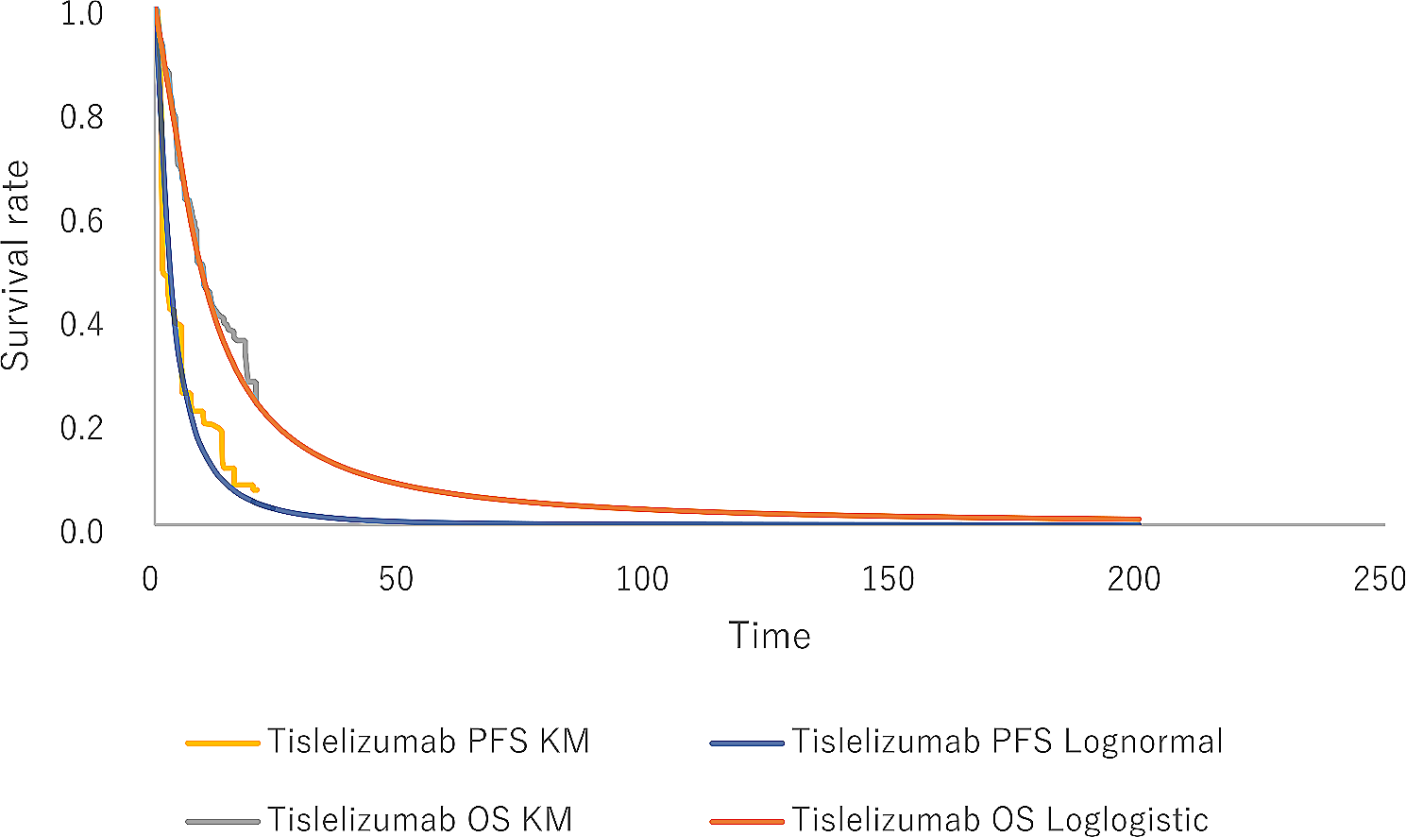
The exploration and fitting of tislelizumab monotherapy OS and PFS

**Fig. 3:**
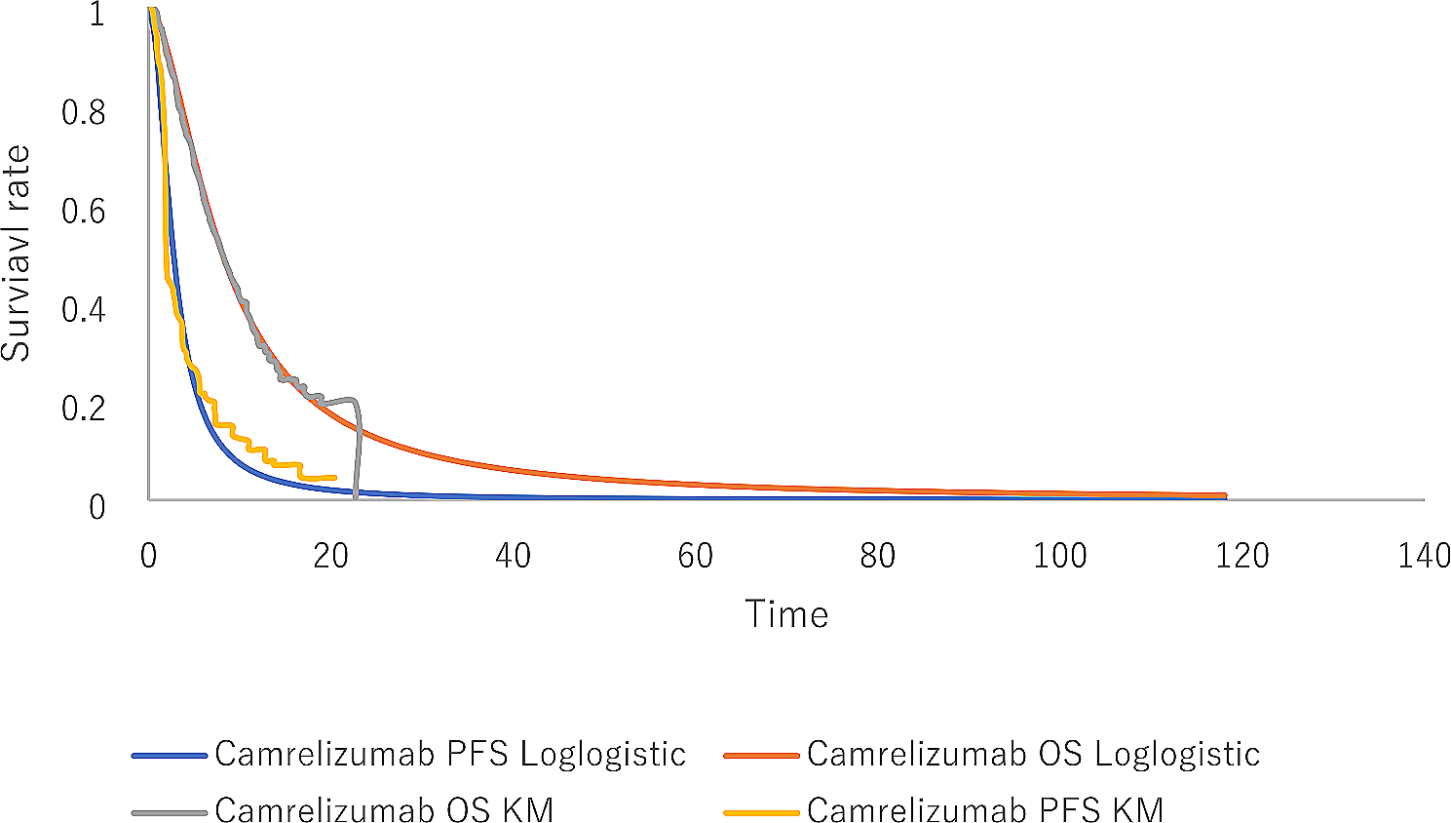
The exploration and fitting of camrelizumab monotherapy OS and PFS

The survival curves of the camrelizumab monotherapy were derived from the published literature [9] and the IPD was reconstructed using the method of *Guyot et al.* study [14]. The results of the parameter fitting are shown in the Supplementary material table S1. Akaike information criterion (AIC), Bayesian information criterion (BIC), visual inspection, and logic error checking were used to evaluate best-fitting parametric distributions. As a result, the best-fitting distribution for PFS data of tislelizumab monotherapy was log-normal distribution, while the best-fitting parametric distributions for OS data of tislelizumab and PFS along with OS data of camrelizumab monotherapy were log-logistic distributions. The fitting and extrapolation results of PFS and OS curves are shown in Figs. 2 and 3.

Only AEs with incidence ≥ 1% and grade ≥ 3 of patients with camrelizumab or tislelizumab were included for this study. AEs to the camrelizumab monotherapy were obtained from ESCORT [9], including somasthenia, diarrhea, hyponatraemia, anemia, lymphopenia, and reactive cutaneous capillary endothelial proliferation (RCCEP). Thereinto, hyponatraemia, anemia and lymphopenia were AEs associated with tislelizumab monotherapy only.

### Cost input

Only direct medical costs were estimated from the perspective of China’s healthcare system including acquisition costs of drugs, subsequent treatment, follow-up visits, disease management, AE management, and end-of-life care.

Drug costs consisted of the costs of tislelizumab and camrelizumab. The prices of tislelizumab and camrelizumab were derived from the cost database available on Menet [15]. Follow-up costs were divided into three parts, including the PF state of the tislelizumab monotherapy, the PF state of the camrelizumab monotherapy, and the PD state, each of which included the cost of contrast computed tomography (CT), routine blood tests, blood chemistry, and routine urine tests. The frequency of contrast CT (chest and upper abdomen) was once every two months for both the PF state of the tislelizumab monotherapy and camrelizumab monotherapy, while the rest of the program was performed before each treatment. For patients in the PD state, the contrast CT (chest and upper abdomen) was performed once every two months, and the rest of the program was once every three weeks. The cost of disease management was also calculated separately for the PF state of the tislelizumab monotherapy, the PF state of the camrelizumab monotherapy, and the PD state, each of which included the costs of diagnosis, intravenous administration, nursing, and hospitalization. The costs of follow-up visits and disease management were obtained from the medical service price in ten randomly selected regions, namely Shanghai, Tianjin, Guangdong, Shandong, Anhui, Liaoning, Ningxia, Yunnan, Hebei, and Heilongjiang, after a stratified sampling of cities nationwide according to their level of economic development. Subsequent treatment is the administration of a different chemotherapeutic agent after patients progressed on tislelizumab or camrelizumab which was used in the first course of treatment. Since both tislelizumab and camrelizumab are PD-1 inhibitors, have similar mechanisms and both are second-line treatment drugs recommended by CSCO guidelines, they are clinically substitutable. This study, therefore, assumed that the subsequent treatment after disease progression for the camrelizumab group is consistent with that of the tislelizumab group, based on expert opinion. The subsequent treatment pattern after progression of tislelizumab was derived from IPD from RATIONALE 302, and only regimens with greater than 5% patient use were included in this study, as shown in Table 3.

**Table 3:** Subsequent treatment options for tislelizumab group

Subsequent treatment	Proportion of patients
Anrotinib hydrochloride	9.6%
Apatinib mesylate	8.9%
Paclitaxel	8.9%
Docetaxel	7.5%
Gemeracil or Otilacil potassium or Tegafur (Tegeo tablets)	7.5%
Irinotecan	5.5%
Cisplatin	8.2%

Only grade 3 or 4 AEs occurring in ≥ 1% of patients were considered in the costs of AE management, including somasthenia, diarrhea, hyponatraemia, anemia, lymphopenia, and reactive cutaneous capillary endothelial proliferation (RCCEP). Since no therapy was clinically essential for non-severe RCCEP, only the cost of severe RCCEP treatment was considered in the model. AEs treatment drugs, days of treatment and costs were from expert opinions except for the duration of RCCEP, which was derived from the camrelizumab package insert (update May 29, 2019). In addition, the cost of end-of-life care was based on published literature [16]. This study assumed that the mean weight of patients was 60 kg and the mean body surface area was 1.6 m^2^ to estimate the dosages of drugs, according to the recommendation from the National Healthcare Security Administration (NMPA) in China. All cost parameters are listed in Table 4.

**Table 4:** Key parameters input

Parameters	Deterministic	Distribution	Low	High	Source
**Cost($)**					
**Unit drug costs ($)**					
Tislelizumab	200.55(per 100 mg)	Constant	160.44	200.55	MENET*
Camrelizumab	404.98(per 200 mg)	Constant	323.98	404.98	MENET*
**Subsequent treatment per cycle($)**					
	721.9	Gamma	675.96	845.46	RATIONALE 302 IPD
**Unit follow-up costs ($)**					
PF state of the tislelizumab (per cycle)	25.45	Gamma	/	/	Expert opinion
PF state of the camrelizumab (per cycle)	29.59	Gamma	/	/	Expert opinion
PD state (per cycle)	25.45	Gamma	/	/	Expert opinion
Contrast CT	25.9	Gamma	10.37	69.16	Health care document**
Blood chemistry	6.43	Gamma	3.96	13.14	Health care document**
Blood routine	1.31	Gamma	0.69	2.35	Health care document**
Urine routine	0.52	Gamma	0.28	0.83	Health care document**
**Unit drug administration costs ($)**					
PF state of the tislelizumab (per cycle)	9.34	Gamma	/	/	Expert opinion
PF state of the camrelizumab (per cycle)	14	Gamma	/	/	Expert opinion
PD state (per cycle)	10.44	Gamma	/	/	Expert opinion
Consulting fee	0.97	Gamma	0.55	3.04	Health care document**
Intravenous injection	0.83	Gamma	0.55	1.66	Health care document**
Hospitalization	3.04	Gamma	1.66	6.64	Health care document**
PF state nursing	1.45	Gamma	0.41	3.04	Health care document**
PD state nursing	3.39	Gamma	3.39	6.22	Health care document**
**Unit AE management costs ($)**					
Somasthenia	106.64	Gamma	0	127.97	Expert opinion
Anemia	467.5	Gamma	86.31	730.29	[34]
Diarrhea	406.4	Gamma	222.96	733.61	Expert opinion
Lymphopenia	82.99	Gamma	3.73	733.61	[34]
Hyponatraemia	326.42	Gamma	14.94	829.88	Expert opinion
RCCEP	276.63	Gamma	8.3	1383.13	[24]
**End-of-life care costs ($)**					
	3949.22	Gamma	3159.38	4739.07	[16]
**Probabilities**					
**Tislelizumab Arm**					
Hyponatraemia	3.10%	Beta	1.38%	5.64%	RATIONALE 302 [8]
Anemia	3.88%	Beta	1.92%	6.67%	RATIONALE 302 [8]
Lymphopenia	3.10%	Beta	1.38%	5.64%	RATIONALE 302 [8]
**Camrelizumab Arm**					
Somasthenia	1.32%	Beta	0.27%	3.15%	ESCORT [9]
Diarrhea	1.32%	Beta	0.27%	3.15%	ESCORT [9]
Hyponatraemia	1.32%	Beta	0.27%	3.15%	ESCORT [9]
Anemia	2.63%	Beta	0.98%	5.07%	ESCORT [9]
Lymphopenia	1.32%	Beta	0.27%	3.15%	ESCORT [9]
RCCEP	79.82%	Beta	74.39%	84.77%	ESCORT [9]
Severe RCCEP	0.44%	Beta	0.01%	1.61%	ESCORT [9]
**Utilities**					
PF state	0.741	Beta	0.593	0.889	[18]
PD state	0.581	Beta	0.465	0.697	[18]
**Disutilities**					
Somasthenia	-0.07	Beta	-0.056	-0.084	[25]
Anemia	-0.07	Beta	-0.06	-0.09	[25]
Diarrhea	-0.07	Beta	-0.056	-0.084	[25]
Lymphopenia	-0.2	Beta	-0.16	-0.24	[24]
Hyponatraemia	0	Beta	0	0	[26]
RCCEP	-0.1	Beta	-0.08	-0.12	[25]
**Discount rate**					
	5%	constant	0%	8%	[13]

PF: progression-free; PD: progressive disease; IPD: individual patient data. *The price of the drug was obtained from MENET: the online price database in China. (https://menet.com.cn). **The price of follow-up and drug administration were obtained from the healthcare document of 10 provinces in China. ***The inclusion criteria of AEs were that the incidence of AEs ≥ 1% and grade ≥ 3

### Utility

Due to the lack of studies using the EuroQol Five Dimensions Questionnaire (EQ-5D) to measure health utility values in second-line ESCC patients directly, this study traced the source of health utility values in published studies of second-line treatment for ESCC and found that the majority health utility values of studies [9, 17–22] (PF = 0.741, PD = 0.581) were derived from a quality-of-life study of ramucirumab plus paclitaxel in patients with previously treated gastric or gastroesophageal junction adenocarcinoma [16]. In that study, the baseline health utility value of patients in the intervention group was 0.74 and the health utility value of patients who discontinued treatment was 0.581 [16]. A study published by the National Institute for Health and Care Excellence (NICE) noted that the baseline health utility value of patients could be used as their PF state utility value and the health utility value for patients who discontinue treatment could be used as their PD state utility value [23]. Therefore, the utility value for this study was 0.741 for the PF state, 0.581 for the PD state, and 0 for death (Table 4).

AE’s disutilities were also considered in the model, which were obtained from the published literature [24–26]. Since RCCEP occurred continuously with the use of camrelizumab, the disutility of camrelizumab during treatment was calculated based on the median duration of RCCEP occurrence in the camrelizumab package insert, rather than just considering the disutility of severe RCCEP. The disutility of RCCEP was assumed to be consistent with that of rash in the Chinese population and the disutility of lymphopenia was assumed to be consistent with that of white blood cell count reduced. The costs and disutilities of AEs were only calculated in the first cycle. Details of incidence rate, disutility values, and sources are listed in Table 4.

### Sensitivity analyses

#### Deterministic sensitivity analyses

To verify the robustness of model results, individual uncertainty parameters were identified, and one-way sensitivity analyses were performed separately within their range of possible variation. The tornado diagram was drawn to identify the factors that have a greater impact on the results. The following key parameters were included in the one-way deterministic sensitivity analyses (DSA): discount rate, costs of drug acquisition, disease management, follow-up, subsequent treatment, and end-of-life care (varied by the standard error, 95% confidence interval), incidence and duration of AEs (varied by 95% confidence interval), utilities and disutilities (varied by 95% confidence interval). Specifically, the price of tislelizumab and camrelizumab was varied from 80 to 100% of the deterministic value because, in China, there is little space for pharmaceutical manufacturers to increase the price of drugs.

#### Probabilistic sensitivity analysis

Probabilistic sensitivity analysis (PSA) was performed using a Monte Carlo simulation with 1,000 iterations. The parametric distribution assumptions were based on the recommendations in *Decision Modelling for Health Economic Evaluation* [27], where the incidence of AEs and utility parameters obeyed the beta distribution, cost parameters and duration of AEs obeyed the gamma distribution. Scatter plots and cost-effectiveness acceptability curves (CEACs) were plotted based on the simulation results to determine the probabilities of being cost-effective for each alternative under different WTP thresholds. In addition, it should be noted that Cholesky decomposition was conducted to take into account the correlation between parameters.

#### Scenario analysis

Since the control group in ESCORT was docetaxel or irinotecan, while that of RATIONALE 302 was paclitaxel, docetaxel, or irinotecan. A scenario analysis was conducted to consider the influence of different control groups on the outcomes. In this scenario, patients with pre-randomized investigator choice of paclitaxel in RATIONALE 302 were excluded and adjustments for patient baseline were performed. The cost-effectiveness of tislelizumab monotherapy in comparison to camrelizumab monotherapy was then evaluated.

## Results

### Base case analysis

The base-case analysis result is presented in Table 5. Tislelizumab monotherapy produced an additional 0.32 LYs and 0.25 QALYs while costing $506 less than camrelizumab monotherapy. The incremental cost-effectiveness ratio (ICER) of tislelizumab monotherapy was -$2,051/QALY compared with camrelizumab monotherapy, indicating that camrelizumab monotherapy was dominated by tislelizumab monotherapy.

**Table 5:** Summary of the cost and health outcomes results

	Tislelizumab monotherapy	Camrelizumab monotherapy
**QALYs**	**0.87**	**0.63**
PF health state	0.33	0.26
PD health state	0.54	0.409
**LYs**	**1.38**	**1.05**
**Total costs**	**$8,499**	**$8,851**
Drug costs	$3,102	$3,698
Disease management costs	$4,603	$4,636
Follow-up visit cost	$610	$492
AE costs	$31	$27
**Incremental costs**	**-$506**	
**Incremental QALYs**	**0.25**	
**Incremental LYs**	**0.32**	
**ICUR**	**-$2,051/QALY**	
**ICER**	**-$1,559/LY**	

QALY: quality-adjusted life year; PF: progression-free; PD: progressive disease; LY: life year; AE: adverse event; ICUR: incremental cost-utility ratio; ICER: incremental cost-effectiveness ratio

### Sensitivity analyses

#### Deterministic sensitivity analyses

**Fig. 4:**
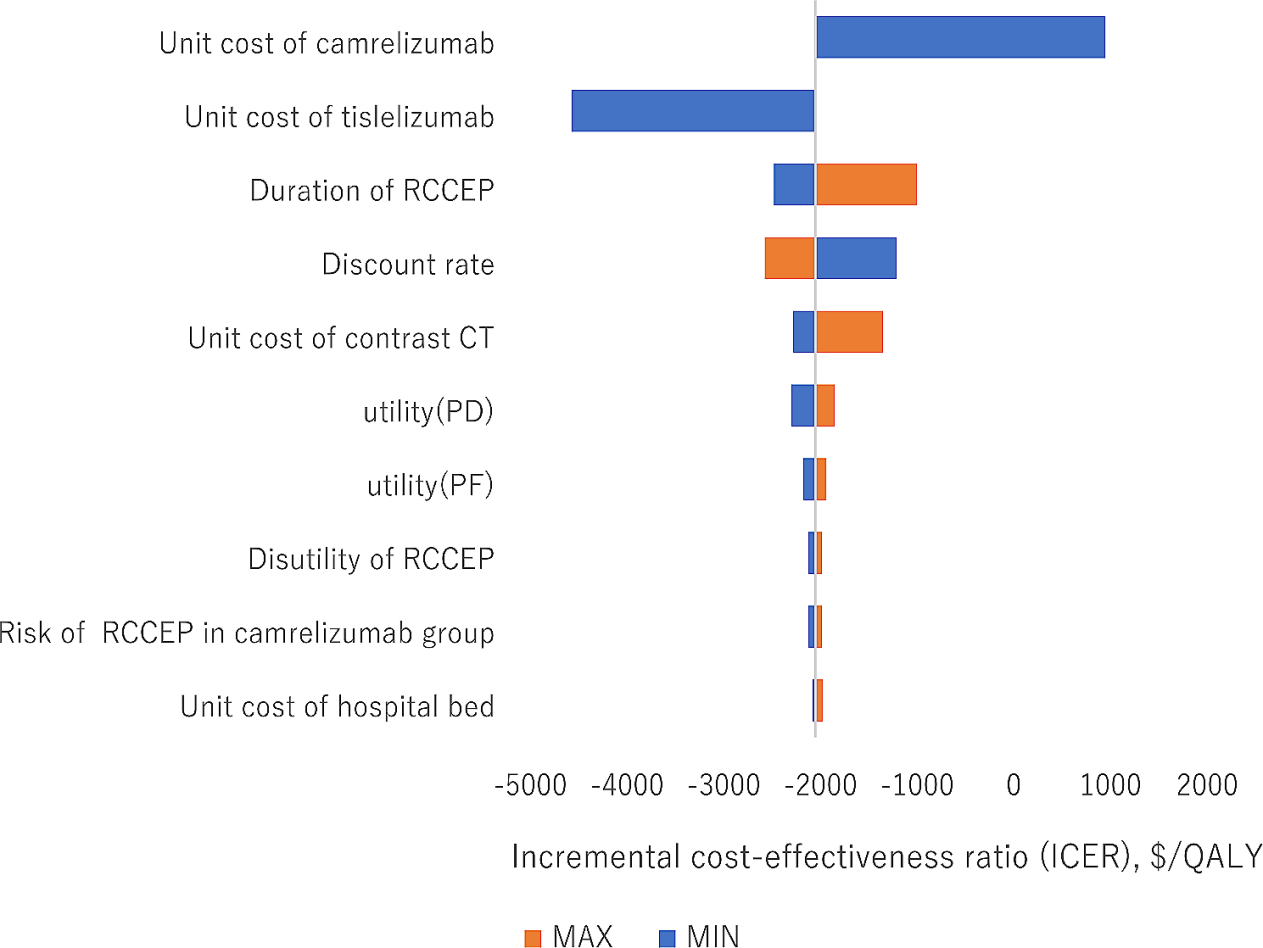
Tornado diagram. PF: progression-free; PD: progressive disease When ICER < 0, tislelizumab is the dominant option compared with camrelizumab

The tornado diagram illustrated the top ten most influential key parameters in one-way sensitivity analyses (Fig. 4). According to the results, the unit cost of camrelizumab, the unit cost of tislelizumab, and the duration of RCCEP were the main driving parameters in the model. The ICUR results from the DSA revealed that tislelizumab monotherapy was consistently more cost-effective than camrelizumab monotherapy. As shown in Fig. 4, the ICER value was most sensitive to the price per unit of camrelizumab and AE’s disutilities had little impact on the outcome.

#### Probabilistic sensitivity analyses

**Fig. 5:**
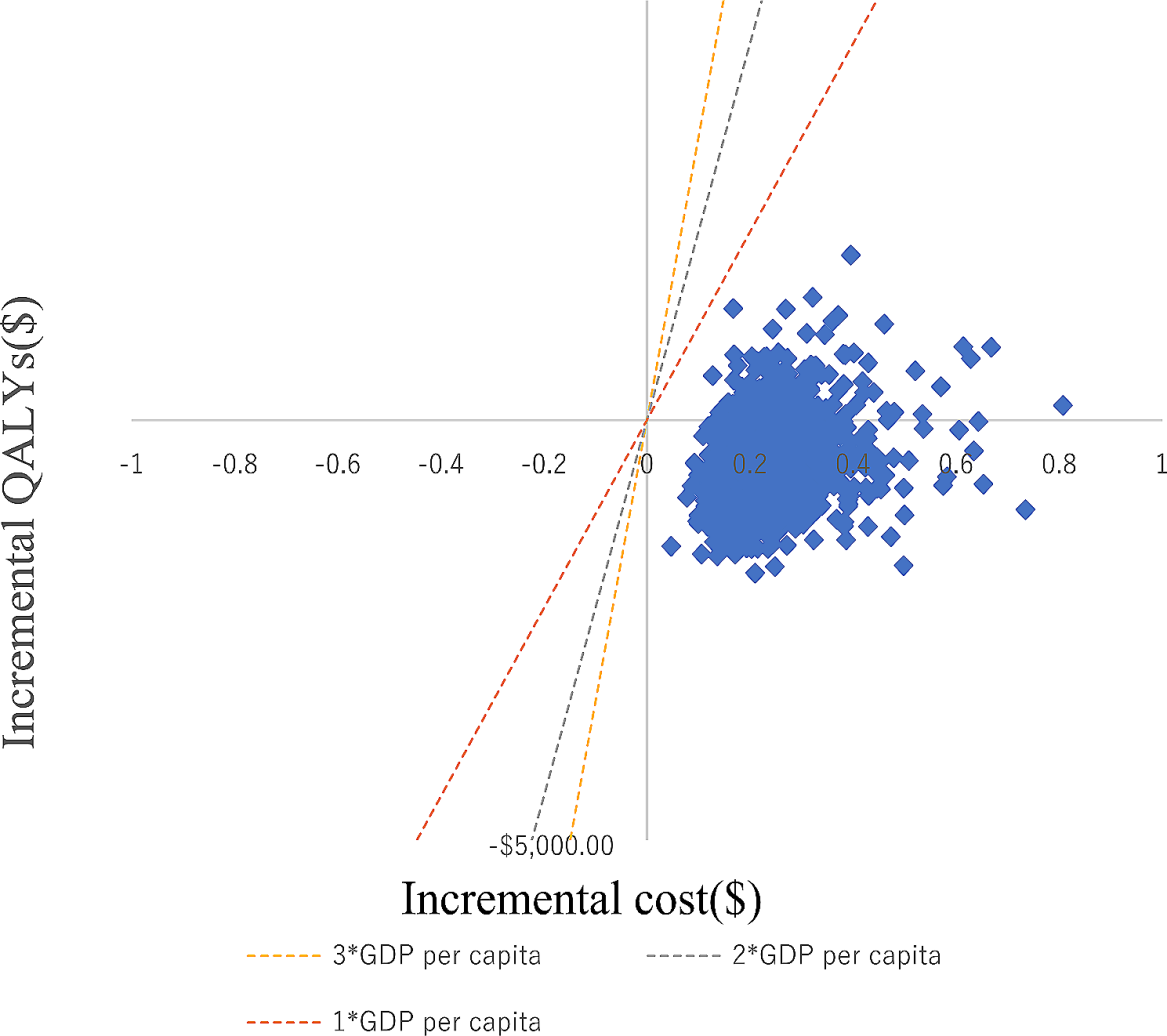
Scatter plot of incremental cost-effectiveness plane

**Fig. 6:**
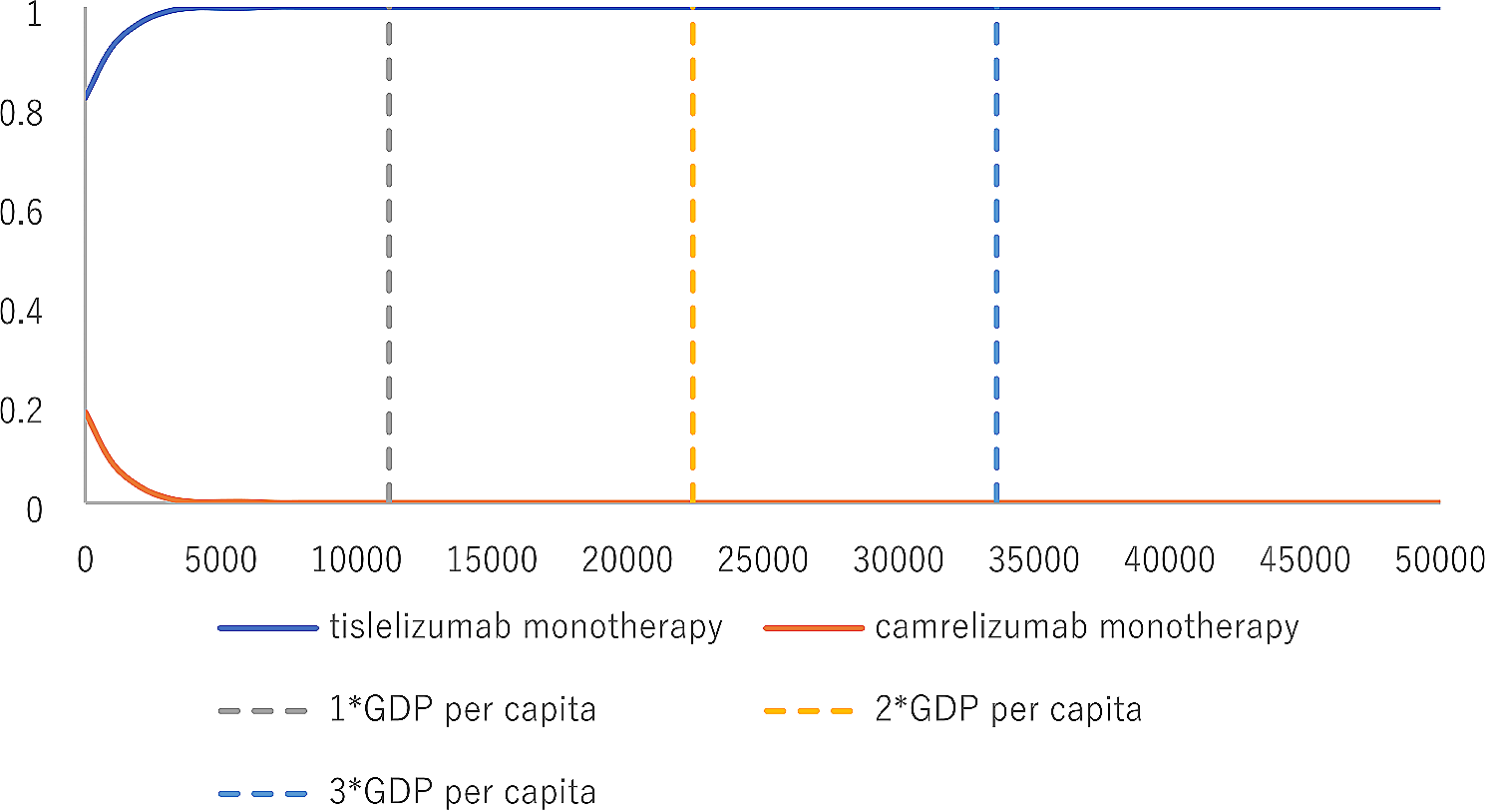
Cost-effectiveness acceptability curve

The results of the probabilistic sensitivity analysis are summarized as a scatterplot and CEACs. The scatterplot showed that most of the iteration results from the PSA fall in the northwest and southwest quadrants (Fig. 5). According to the CEACs, at a WTP threshold of $11,207/QALY∼$33,621/QALY (1–3 GDP per capita in China), the probability that tislelizumab monotherapy was cost-effective compared with camrelizumab monotherapy was almost 100% (Fig. 6).

#### Scenario analysis

The scenario analysis results are shown in Table 6. Over a lifetime horizon, the tislelizumab monotherapy group gained 0.89 QALYs with a cost of $8,499, while the camrelizumab monotherapy group gained 0.63 QALYs with a cost of $8,851. The incremental QALYs and cost for tislelizumab monotherapy against camrelizumab monotherapy were 0.26 QALYs and -$352, respectively, suggesting that tislelizumab monotherapy dominated camrelizumab monotherapy for the second-line treatment of locally advanced or metastatic ESCC in China.

**Table 6:** The results of scenario analysis

	Tislelizumab monotherapy	Camrelizumab monotherapy
**QALYs**	**0.89**	**0.63**
PF health state	0.35	0.26
PD health state	0.54	0.409
**LYs**	**1.40**	**1.05**
Total costs	$8,499	$8,851
Drug costs	$3,247	$3,698
Disease management costs	$4,602	$4,636
Follow-up visit cost	$619	$492
AE costs	$31	$27
**Incremental costs**	**-$352**	
**Incremental QALYs**	**0.26**	
**Incremental LYs**	**0.35**	
**ICUR**	**-$1,344/QALY**	
**ICER**	**-$1,020/LY**	

## Discussion

The study evaluated the cost-effectiveness of tislelizumab monotherapy versus camrelizumab monotherapy in patients with locally advanced and metastatic ESCC who have failed after first-line chemotherapy treatment from the perspective of China’s healthcare system based on the IPD from RATIONALE 302, which was weighted to balance the reported characteristics of patients in ESCORT using MAIC. Our analysis encompassed a comprehensive assessment of various parameters, including clinical data, costs, and health-related quality of life, providing valuable insights into the economic considerations of these treatment choices for ESCC patients. The base-case results showed that tislelizumab monotherapy incurred a lower lifetime cost and yielded higher QALYs, which resulted in an ICER of -$2,051/QALY. It implied that tislelizumab monotherapy was dominant versus camrelizumab monotherapy under the recommended thresholds of 1 to 3 times China’s GDP per capita in 2021. Sensitivity analyses, both DSA and PSA, further supported the robustness of our study results. Tornado diagrams highlighted key factors influencing the model outcomes, particularly emphasizing the duration of the adverse events of rash and diarrhea, and the unit costs of Tislelizumab and Camrelizumab. CEAC demonstrated a high probability of economic acceptability for Tislelizumab monotherapy over Camrelizumab at various WTP thresholds.

Several studies had been conducted to assess the cost-effectiveness of second line treatments for ESCC, and most focused on the cost-effectiveness of camrelizumab. Cai et al. used a PSM to compare the cost-effectiveness of camrelizumab, docetaxel or irinotecan from the societal perspective of China, which had a shorter time horizon (10 years) and longer cycle length (1 month) than this study [28]. In that study, camrelizumab monotherapy was cost-effective compared with chemotherapy group. Lin et al. published a similar PSM-based study but with shorter time horizon (two years) and got similar findings with Cai et al [28, 29]. In addition, Yang et al. used the Markov model to evaluate the cost-effectiveness of camrelizumab, docetaxel or irinotecan from the perspective of the China’s healthcare system, which also indicated that camrelizumab was cost-effective than the chemotherapy group [30]. This showed that the choice of model type and the perspective of the studies did not affect the results of the cost-effectiveness of camrelizumab versus the chemotherapy group. Shi et al. used a PSM to compare the cost-effectiveness of tislelizumab versus chemotherapy as a second-line treatment for advanced or metastatic ESCC in China and found that tislelizumab therapy was highly cost-effective compared with chemotherapy therapy [31]. It implied that tislelizumab could be a promising strategy for treating ESCC patients in the setting of China. In a summary, all of the studies above chose chemotherapy as the comparator. To the best of our knowledge, this is the first study to compare the cost-effectiveness of tislelizumab versus camrelizumab as a second-line treatment for advanced or metastatic ESCC in China. The previous studies concluded that camrelizumab was cost-effective, however, this study proved that tislelizumab had better results than camrelizumab.

In addition, our study is important and instructive because it draws attention to some issues that should be heeded in the cost-effectiveness analyses of anti-oncology drugs when using indirect comparison methods, especially the solution to the inaccessibility of some key data. Due to the lack of head-to-head clinical trials of the tislelizumab monotherapy and the camrelizumab monotherapy, data were obtained from the RATIONALE 302 and the ESCORT, respectively, and the anchor MAIC method was used to adjust the baseline characteristics of the two groups for indirect comparisons, thereby reducing the bias associated with indirect comparisons. The indirect comparisons and the PSM were based on IPD from the RATIONALE 302, and the data were of high quality. For data that were difficult to obtain from the literature, detailed interviews were conducted with experts who had extensive clinical experience in the treatment of locally advanced or metastatic ESCC.

We conducted a scenario analysis to account for the influence of different control groups, particularly the choice between paclitaxel and docetaxel, in RATIONALE 302 and ESCORT. In this scenario, we excluded patients with pre-randomized investigator choice of paclitaxel in RATIONALE 302, performed adjustments for patient baseline characteristics, and re-evaluated the cost-effectiveness of tislelizumab monotherapy against camrelizumab monotherapy. The results of this scenario analysis reaffirmed the dominance of tislelizumab monotherapy.

There exist several limitations that should be noted in our work. To address concerns about the validity of our model, we employed various validation techniques. This included expert consultations during model development, expert meetings to validate results, and thorough comparisons with clinical trial data. Sensitivity analyses, both deterministic and probabilistic, confirmed the model’s internal validity. However, it is crucial to acknowledge certain limitations in our study. Firstly, the utility values used were derived from studies on other cancer types due to the lack of ESCC-specific data, potentially introducing some uncertainty into our results. However, the utility values from this study have been cited in several published ESCC-related economic evaluations [9, 19, 22, 28, 32, 33]. Additionally, we extrapolated hazard ratios for survival outcomes as the proportional hazard assumption was not met in the available data. Since the PFS curves of tislelizumab before and after MAIC adjustment relative to the chemotherapy group and the PFS curves of camrelizumab relative to the chemotherapy group did not meet the PH assumption, the HR values of both relative to the chemotherapy group were not used for the calculation of this study, but were extrapolated by fitting the parameters to their respective IPDs, which may have biased the results to some extent. Furthermore, the indirect comparison approach necessitated some assumptions and adjustments in combining data from the RATIONALE 302 and ESCORT trials, potentially introducing bias despite our best efforts to minimize it RATIONALE 302 is a randomized, open-label, multicenter, global phase 3 clinical trial comparing the efficacy and safety between tislelizumab monotherapy and ICC as second-line treatment in locally advanced or metastatic ESCC, whereas ESCORT targets only Chinese patients. When adopting the MAIC method, in order to match the ESCORT patients, RATIONALE302 only used the Chinese subpopulation (including Taiwan), while excluding patients older than 75 years or those with brain metastases, resulting in a degree of bias in the baseline adjustment results. Besides, since the lack of studies using the EQ-5D to measure health utility values in second-line ESCC patients directly, most of the AE disutilities considered in this study were derived from lung cancer-related utility studies, which may not truly reflect the impact of AE on the health utility of ESCC patients, and some of the AE treatment costs were derived from published literature, which may deviate from clinical. Furthermore, the disutility of RCCEP in this study was assumed to be consistent with that of rash, however, RCCEP may cause skin breakdown and bleeding, and grade 3 or above RCCEP may be associated with skin infections requiring hospitalization, so the disutility of RCCEP may be higher than that of rash, and this assumption may underestimate the impact on patient health and therefore the cost-effectiveness of tislelizumab monotherapy.

Our findings have implications for healthcare policy and resource allocation in China. The cost-effectiveness of tislelizumab as a second-line treatment for ESCC can guide healthcare decision-makers in prioritizing resources and improving patient outcomes in a financially sustainable manner. In future research, incorporating patients, healthcare professionals, and other stakeholders directly into the study design process may provide additional perspectives and insights into priorities and preferences within the specific healthcare context. This comprehensive approach could better meet patients’ needs and guide decision-making more effectively, ensuring that future research aligns with the preferences of those directly involved.

## Conclusion

According to the base-case analysis and the sensitivity analyses, tislelizumab monotherapy is cost-effective compared with camrelizumab monotherapy in China as the second-line treatment for locally advanced or metastatic ESCC patients from the perspective of China’s healthcare system.

### Electronic supplementary material

Below is the link to the electronic supplementary material.

Supplementary Material 1

## Data Availability

All data generated or analysed during this study are included in this published article and its supplementary information files.
