# Supplementary material for: Cost-effectiveness analysis of tislelizumab vs. camrelizumab for the treatment of second-line locally advanced or metastatic esophageal squamous cell carcinoma
Source: BMC Health Serv Res. 2024 May 29;24:676. doi: 10.1186/s12913-024-11142-5 (PMC11134889; doi:10.1186/s12913-024-11142-5)
Supplement: Supplementary file 1 — Supplementary Material 1 [file 12913_2024_11142_MOESM1_ESM.docx]

**Figure S1 Unweighted PH assumption for RATIONALE 302, tislelizumab relative to the chemotherapy group**


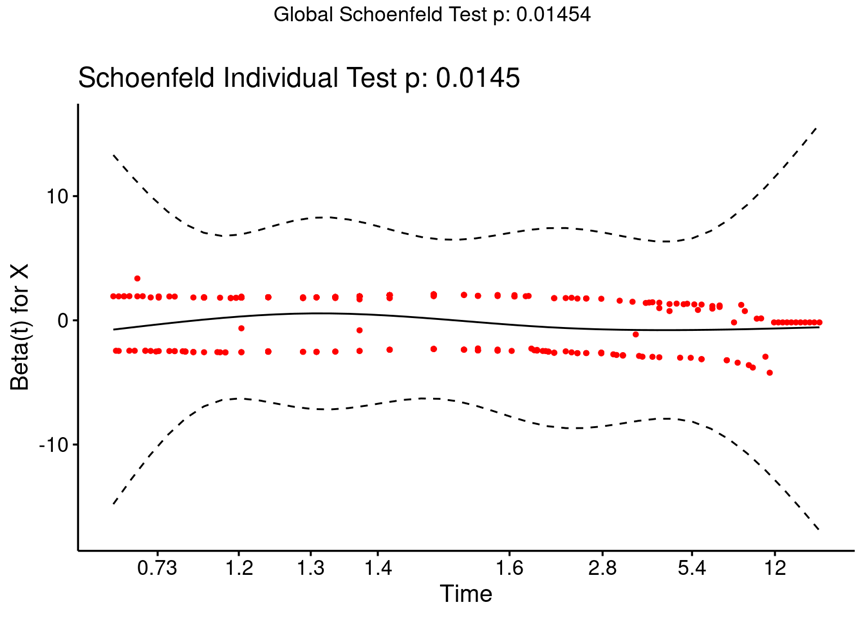


**Figure S2 Weighted PH assumption for RATIONALE 302, tislelizumab relative to the chemotherapy group**


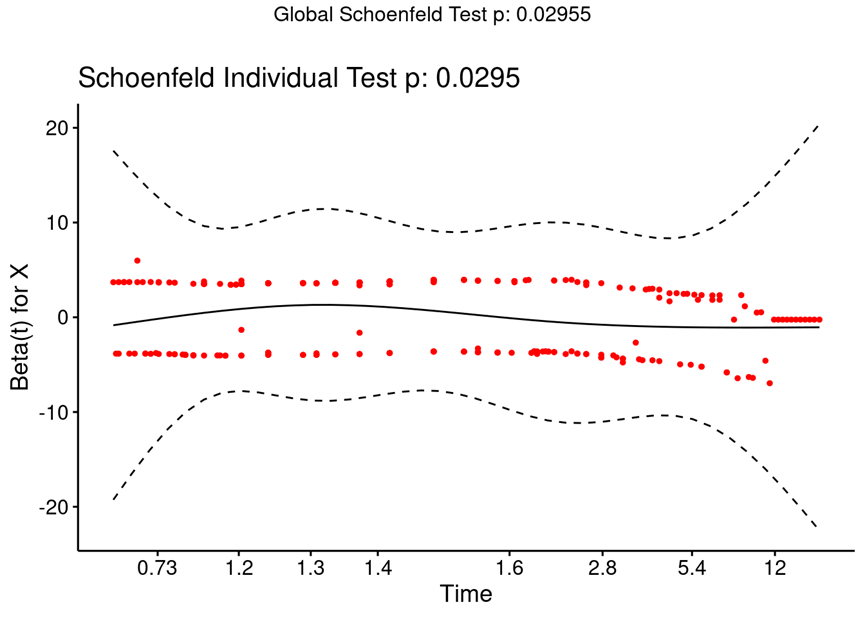


**Table S1 Statistical testing of tislelizumab/camrelizumab monotherapy OS and PFS**

| **Statistical testing** | **Weibull** | **gamma** | **exponential** | **log-logistic** | **log-normal** | **Gompertz** | |
| --- | --- | --- | --- | --- | --- | --- | --- |
| **OS data of tislelizumab monotherapy** | | | | | | |  |
| AIC | 539.7 | 539.5 | 538.6 | 542.3 | 542.5 | 539.8 | |
| BIC | 545.8 | 545.6 | 541.6 | 548.4 | 548.6 | 545.9 | |
| Parameter | shape=1.1002  scale=14.1609 | shape=1.1670 rate=0.0849 | rate=0.0713 | shape=1.4638 scale=9.2488 | meanlog=2.1832 sdlog=1.1903 | shape=0.0146 rate=0.0623 | |
| **PFS data of tislelizumab monotherapy** | | | | | | | |
| AIC | 394.0 | 396.1 | 394.5 | 373.4 | 370.9 | 384.9 | |
| BIC | 400.1 | 402.2 | 397.5 | 379.5 | 377.0 | 391.0 | |
| Parameter | shape=0.8755 scale=5.1761 | shape=0.9153 rate=0.1677 | rate=0.1853 | shape=1.4948 scale=2.6255 | meanlog=1.0745 sdlog=1.1338 | shape=-0.0901 rate=0.2767 | |
| **OS data of camrelizumab monotherapy** | | | | | | | |
| AIC | 1182.0 | 1177.9 | 1191.1 | 1169.5 | 1168.2 | 1191.6 | |
| BIC | 1188.9 | 1184.7 | 1194.5 | 1176.3 | 1175.1 | 1198.5 | |
| Parameter | shape=1.2512 scale=11.8067 | shape=1.4929 rate=0.1350 | rate=0.0840 | shape=1.7145 scale=8.0690 | meanlog=2.0846 sdlog=1.0030 | shape=0.0189 rate=0.0743 | |
| **PFS data of camrelizumab monotherapy** | | | | | | | |
| AIC | 950.3 | 942.8 | 950.7 | 891.3 | 894.8 | 947.6 | |
| BIC | 957.1 | 949.7 | 954.1 | 898.2 | 901.6 | 954.5 | |
| Parameter | shape=1.0877 scale=4.6829 | shape=1.3423 rate=0.3010 | rate=0.2180 | shape=1.9470 scale=2.7334 | meanlog=1.0795 sdlog=0.9120 | shape=-0.0441 rate=0.2563 | |

lIST OF IECS OR IRBS AND REPRESENTATIVE WRITTEN INFORMATION FOR PATIENT AND SAMPLE CONSENT FORM

Table S2: List of Ethics Committees

| Site No. | Investigator Name | Name/Address of Ethics Committee | Name of Chairman |
| --- | --- | --- | --- |
| 086001 | Lin Shen | Medical Ethics Committee of Beijing Cancer Hospital / Beijing No.52, Fu-Cheng Road, Haidian District, Beijing, P.R., China | Li Jie  Hao Chunyi |
| 086003 | Kuaile Zhao | EC of Fudan University Shanghai Cancer Center / No.270, Dong’an Road, Shanghai, China | Chen Zhen |
| 086006 | Jufeng Wang | Medical Ethics Committee of Henan Cancer Hospital / No.127, Dongming Road , Zhengzhou, Henan, China | Song Yongping  Zhang Wenzhou |
| 086007 | Yongqian Shu | EC of The First Affiliated Hospital with Nanjing Medical University / No.300 Guangzhou road, Gulou District, Nanjing, Jiangsu, China | Zhang Fumin |
| 086009 | Lin Zhao | EC of Peking Union Medical College Hospital / No.41 Damucang Hutong, Xicheng District, Beijing, China | Zhang Fengchun |
| 086012 | Wei Li | The IRB of The First hospital of Jilin University / No.71 Xinmin Street, Changchun, China | Niu Junqi  Tan Yuquan |
| 086014 | Yulong Zheng | EC of The First Affiliated Hospital of Zhejiang University / 79 Qingchun Road, Hangzhou, Zhejiang, China | Hu Shenjiang |
| 086020 | Longzhen Zhang | The Clinical Research Ethnics Committee of The Affiliated Hospital of Xuzhou Medical University / No.99 of Huaihai west road, Xuzhou, Jiangsu, China | Xu Tie |
| 086021 | Wei Ren | Medical Ethics Committee of Nanjing Drum Tower Hospital, the Affiliated Hospital of Nanjing University Medical School / No.321, Zhongshan Road, Nanjing City, Jiangsu Province, China | He Zhongzheng |
| 086022 | Yuxian Bai | Harbin Medical University Cancer Hospital Ethics Committee / No. 150 Haping Road, Nangang district, Harbin, Heilongjiang, China | Zhao Changhong |
| 086023 | Shubin Wang | Ethics Committee of Peking University Shenzhen Hospital / Area A, 14th floor, Internal Medicine Inpatient Building, No.1120, Lianhua Road, Futian District, Shenzhen, Guangdong, China | Wang Tao |
| 086024 | Da Jiang | The IRB of Fourth Hospital of Hebei Medical University / No. 12, Jiankang Road, Shijiazhuang, Hebei, China | Shan Baoen  Wang Guiying  He Hongtao |
| 086027 | Xinmin Yu | EC of Zhejiang Cancer Hospital/No.1, Banshandong Road, Gongshu District, Hangzhou, Zhejiang, China | Ge Minghua  Cheng Xiangdong |
| 086031 | Zhendong Chen | EC of The Second Hospital of Anhui Medical University / No. 678 Furong Road, Economic and Technological Development Zone, Hefei, China | Zhao Hui |
| 086036 | Zhiyong He | EC of Fujian Cancer Hospital / No.420, Fuma Road, Jin’an District, Fuzhou, Fujian, China | Wu Hui |
| 086045 | Guohua Yu | Drug Clinical Trial Ethics Committee of Weifang People's Hospital / No.151, Guangwen Street, Kuiwen district, Weifang city, Shandong Province, China | Wang Bingwu |
| 086049 | Jian-Hua Chen | EC of Hunan Cancer Hospital / No.283 Tong Zi Po Road, Changsha, Hunan, China | Yu Huayue |
| 086053 | Ping Lu | Ethics Committee of The First Affiliated Hospital of Xinxiang Medical University / No.88 Jiankang Road, Weihui, Xinxiang, Henan, China | Song Jinggui |
| 086054 | Xi Shi | EC of The First Affiliated Hospital of Fujian Medical University / No. 20, Chazhong Road, Taijiang District, Fuzhou, Fujian, China | Yang Liyong  Ou Qishui |
| 086055 | Sheng Hu | EC of Hubei Cancer Hospital / No.116 Zhuodaoquannan Road, Hongshan District, Wuhan, Hubei, China | Wu Xinhong |
| 086056 | Shirong Cai | The Ethics Committee of Clinical Drugs, Devices and New Medical Technology of the First Affiliated Hospital of Sun Yat-sen University / Room 110, Longzhu Building 1st floor, No. 5 Zhusigangerma Road, Guangzhou, Guangdong Province, China | Yan Churong |
| 086057 | Tienan Yi | Xiangyang Central Hospital Ethics Committee / No.136, Jingzhou Street, Xiangyang, Hubei, China | He Xiaoming  Mao Chun |
| 086058 | Jianhua Shi | Ethics Committee of Linyi Cancer Hospital /  The intersection of Zhicheng Road and Zhongsheng Street, Hedong District, Linyi City, Shandong Province, China (New). No.6, Lingyuan East Road, Lanshan District, Linyi City, Shandong Province, China (Old) | Li Yanping |
| 086059 | Yi Jiang | Medical Ethics Committee of Cancer Hospital of Shantou University Medical College/ No.7 Raoping Load, Shantou, Guangdong Province, China | Qiu Weili |
| 086060 | Qing Bi | EC of Yunnan Cancer Hospital / No.519, Kunzhou Road, Xishan District, Kunming, Yunnan, China | Wu Hongming |
| 086063 | Xueyi Zhou | EC of Huai’an Second Peoples’s Hospital / No.62 Huaihai South Road, Huaian, Jiangsu, China | Xu Jifan |
| 086064 | Aimin Zang | IRB of Affiliated Hospital of Hebei University / No.212 Yuhua East Road, Baoding, Hebei, China | Chen Bing |
| 086065 | Bing Xia | EC of Hangzhou First People’s Hospital / No.261, Huansha Road, Shangcheng District, Hangzhou, Zhejiang, China | Huang Jinyu |
| 086066 | Junping Wang | The IRB of Shanxi Provincial People’s Hospital / No.29, Shuangtasi Street, Taiyuan, Shanxi, China | Huang Bo |
| 086067 | Honglin Hu | Sichuan Academy Of Medical Sciences· Sichuan Provincial People’s Hospital, Ethics Committee For Clinical Trials Of Medicines And Medical Devices / No.32 West of Second 2, 1st Ring Rd, Chengdu, Sichuan, China | Han Shengxi |
| 086068 | Qi Luo | Ethics Committee of The First Affiliated Hospital of Xiamen University / 12th Floor, Hongquan Building, The First Affiliated Hospital of Xiamen University, No.55 Zhenhai Road, Siming District, Xiamen, Fujian, China | Wang Zhanxiang /Lin Mingzhu |
| 086083 | Zhijun Wu (Formerly: 20190121-20200117)  Lei Yang (Currently: Since 20200117) | Ethics Committee of Nantong Tumor Hospital / No.30 Tongyang North Road, Pingchao Town, Tongzhou District, Nantong, Jiangsu, China | Shi Minxin |
| 086084 | Zhiping Li | EC of Jiangxi Province Cancer Hospital / No.519, Beijing Dong Road, Nanchang, Jiangxi, China | Xu Rengen |
| 086091 | Xiaoyan Lin | EC of Fujian Medical University Union Hospital / NO.29 Xinquan Road, Gulou District, Fuzhou, China | Liu Libin |
| 086102 | Zuoxing Niu | Drug Clinical Trial Ethics Committee of Shandong Cancer Hospital (Old) Ethics Committee of Shandong Cancer Hospital & Institute (New) / No.440, Jiyan Road, Huaiyin District, Jinan City, Shandong Province, China | Yu Jinming |
| 086103 | Feng Wang | Research and Clinical Trial Ethics Committee of The First Affiliated Hospital of Zhengzhou University / No.43, Daxue Road, Zhengzhou, Henan, China (New) No.1, Jianshe East Road, Erqi District, Zhengzhou, Henan, China (Old) | Sun Yingpu |
| 086110 | Yanhong Deng | Ethics Committee of the Sixth Affiliated Hospital of Sun Yat-sen University / No. 26 Erheng Road, Yuancun, Tianhe District, Guangzhou, Guangdong Province, China | Wu Xiaojian |
| 086113 | Bangwei Cao | EC of Beijing Friendship Hospital, Capital Medical University / No.95 Yong’an Road, Xicheng District, Beijing, China | Zhang Jian  Xie Miaorong |
| 086117 | Jun Wu | EC of The first people’s Hospital of Changzhou / No.185, Juqian Street, Changzhou, Jiangsu, China | Zhang Xiaoying |
| 086165 | Shuqun Zhang | The Second Affiliated Hospital of Xi'an Jiaotong University Ethics Committee / No.30, Huangcheng West Road, Xi 'an, Shaanxi, China | Sun Xiuzhen |
| 081001 | Taroh Satoh | Osaka University Hospital - Institutional Review Board / 2-15 Yamadaoka Suita Osaka 565-0871 JAPAN | Haruhiko Kishima |
| 081003 | Hiroki Hara | Saitama Cancer Center - IRB/IEC / 780 Komuro, Ina-machi Kitaadachi-gun Saitama 362-0806　JAPAN | Hirofumi Kobayashi |
| 081004 | Keiko Minashi | Chiba Cancer Center - Institutional Review Board / 666-2 Nitona-cho, Chuo-ku Chiba Chiba 260-8717 JAPAN | Hiroshi Ishii |
| 081009 | Takashi Kojima | National Cancer Center Hospital Local IRB/EC / 5-1-1 Tsukiji Chuo-ku Tokyo 104-0045 JAPAN | Noboru Yamamoto |
| 081015 | Ken Kato | National Cancer Center Hospital - Local IRB/EC / 5-1-1 Tsukiji Chuo-ku Tokyo 104-0045 JAPAN | Noboru Yamamoto |
| 081017 | Satoru Motoyama | Akita University Hospital - Institutional Review Board / 44-2 Hasunuma, Hiroomote Akita Akita 010-8543 JAPAN | Katsunori Iijima |
| 081022 | Takahiro Tsushima | Shizuoka Cancer Center - Institutional Review Board / 1007 Shimonagakubo, Nagaizumi-cho Sunto-gun Shizuoka 411-8777 JAPAN | Noriyuki Masuda |
| 081023 | Yu Sunakawa | St. Marianna University School of Medicine Hospital Review Board/ 2-16-1 Sugao, Miyamae-ku Kawasaki Kanagawa 216-8511 JAPAN | Kazuo Yudoh |
| 081026 | Morihito Okada | Hiroshima University Hospital - IRB/IEC / 1-2-3 Kasumi, Minami-ku Hiroshima Hirosima 734-8551 JAPAN | Hiroaki Matsuo |
| 081029 | Manabu Muto | Kyoto University Hospital - IRB/IEC / 54 Shogoin-kawahara-cho Sakyo-ku Kyoto-shi Kyoto 606-8507 JAPAN | Hiroshi Kawakami |
| 081030 | Masaru Morita | National Kyushu Cancer Center Institutional Review Board / 3-1-1 Notame, Minami-ku Fukuoka Fukuoka 811-1395 JAPAN | Taito Esaki |
| 081031 | Ryu Ishihara | Osaka International Cancer Institute - IRB / 3-1-69 Otemae Chuo-ku Osaka-shi Osaka 541-8567 JAPAN | Ryu Ishihara |
| 081032 | Tomohiro Nishina | Shikoku Cancer Center - Irb / 160 Kou, Minami-umemoto-machi Matsuyama-shi Ehime 791-0280 JAPAN | Toshiyuki Kozuki |
| 081033 | Akihito Tsuji | Kagawa University Hospital – IRB / 1750-1 Miki-cho Ikenobe Kita-gun Kagawa 761-0793 JAPAN | Hideto Yokoi |
| 081034 | Hisateru Yasui | Kobe City Medical Center General Hospital - IRB / 2-1-1, Minatojima-Minami-machi, Chuo-ku Kobe 650-0047 JAPAN | Yasushi Naito |
| 081035 | Masahiro Tsuda | Hyogo Cancer Center - Irb / 13-70 Kitaoji-Cho Akashi Hyogo 673-8558 JAPAN | Miyako Satouchi |
| 081036 | Yuichi Shibuya | Kochi Health Sciences Center - IRB / 2125-1 Ike , Kochis-shi, Kochi 781-8555 JAPAN | Akihito Nishioka |
| 081037 | Masahiro Goto | Osaka Medical College Hospital – IRB / 2-7 Daigaku-machi Takatsuki, Osaka 569-8686 JAPAN | Ryuichi Saura |
| 081038 | Takao Tamura | Kindai University Nara Hospital - IRB / 1248-1 Otodacho Ikoma Nara 630-0293 JAPAN | Masato Muraki |
| 081048 | Keisho Chin | The Cancer Institute Hospital of JFCR - Institutional Review Board / 3-8-31 Ariake Koto-Ku Tokyo 135-8550 JAPAN | Manabu Ohashi |
| 082015 | Young Saing Kim | Gachon University Gil Medical Center - IRB / 21 Namdong-daero 774beon-gil Namdong-gu Incheon 21565 KOREA, REPUBLIC OF | NA in Korea |
| 082016 | Sang Cheul Oh | Korea University Guro Hospital - IRB / 148 Gurodong-ro Guro-gu Seoul 08308 KOREA, REPUBLIC OF | NA in Korea |
| 082031 | Jong-Seok Lee | Seoul National University Bundang Hospital / 82 Gumi-ro, 173 beon -gil Bundang-gu Seongnam-si Gyeonggido [Kyonggi-do] 13620 KOREA, REPUBLIC OF | NA in Korea |
| 082032 | Sung Bae Kim | Asan Medical Center - Oncology / 88 Olympic-ro 43-gil Songpa-Gu,Asan Medical Center, 88 Seoul Seoul Teugbyeolsi [Seoul-T'ukp 05505 KOREA, REPUBLIC OF | NA in Korea |
| 082034 | Ik Joo Chung | Chonnam National University Hwasun Hospital / 322 Seoyang-ro Hwasun-eup Hwasun-gun Jeollanam-do 58128 KOREA, REPUBLIC OF | NA in Korea |
| 082035 | Jong-Mu Sun | Samsung Medical Center - IRB / 81 Irwon-ro Gangnam-gu Seoul 06351 KOREA, REPUBLIC OF | NA in Korea |
| 886004 | Chien-Liang Lin | Chi Mei Medical Center - YongKang - IRB/IEC / No.901, Zhonghua Rd. Yongkang Tainan 71004 TAIWAN | Chung-Hsi Hsing |
| 886006 | Chuan-Cheng Wang | Changhua Christian Hospital - Institutional Review Board / No. 135, Nanxiao Street Changhua City, Changhua County 500, Taiwan (R.O.C.) | KunTu Yeh |
| 886007 | Shau-Hsuan Li | Chang Gung Medical Foundation - Kaohsiung Chang Gung Memorial Hospital - IRB/IEC / 199, Tung Hwa North Road, Taipei, Taiwan, 10507 | Tsang-Tang Hsieh |
| 886009 | Wei-Yu Chen | Chi Mei Medical Center - YongKang - IRB/IEC / No.901, Zhonghua Rd. Yongkang Tainan 71004 TAIWAN | Chung-Hsi Hsing |
| 886041 | Chih-Hung Hsu | National Taiwan University Hospital - IRB/IEC / No 1 Chung-Te Street, Floor 2 Taipei Taipei 10048 TAIWAN (Actual address) National Taiwan University Hospital, 7, Chung-Shan South Road, Taipei, Taiwan 100, ROC (This is the address on IRB approval letter) | Daniel Fu-Chang Tsai |
| 886045 | Yee Chao | Taipei Veterans General Hospital - Irb / 201, Shih-Pai Road Sec. 2, Taipei, TAIWAN 11217, Republic of China | Shinn-Jang Hwang(previous)  Shih-Ann Chen(current) |
| 886048 | Chen-Yuan Lin | China Medical University Hospital - IRB/IEC - Taichung / 6th Conference Room, B1, First Medical Building, No. 2, Yu-De Rd., North area Taichung 40447 TAIWAN (Actual address) China Medical University Hospital, 2 Yude Road, Taichung, 20227, Taiwan (R.O.C) (This is the address on IRB approval letter) | Martin M-T Fuh |
| 032008 | Joëlle Collignon | Comité d'étique Hospitalo-Facultaire Universitaire de Liège - Domaine Universitaire du Sart Tilman / Bâtiment B 35 Liège Liège 4000, BELGIUM | Prof. Vincent Seutin |
| 032009 | Pieter-Jan Albert René Cuyle | Commissie voor Medische Ethiek Imelda Ziekenhuis / Imeldalaan 9 Bonheiden Antwerpen 2820, BELGIUM | Dr. Stijn Gysenbergs |
| 032010 | Marc Van den Eynde | Comité d'Ethique Hospitalo-Facultaire Cliniques Universitaires Saint-Luc / Promenade de l'Alma 51 bte B1.43.03 Bruxelles Brussels Capital Region 1200, BELGIUM | Dr. Jean-Marie Maloteaux |
| 032012 | Marc Peeters | Ethisch Comité UZ Antwerpen / Drie Eikenstraat 655 Edegem Antwerpen 2650, BELGIUM | Prof. Peter Michielsen |
| 032013 | Eric Van Cutsem | Ethische Commissie Onderzoek UZ/KU Leuven - Campus Gasthuisberg / Herestraat 49 / B 3000 Leuven, BELGIUM | Prof. Minne Casteels |
| 033004 | Michel Ducreux | Comité de Protection des Personnes Est IV - 1, Place de l’Hôpital -67091 STRASBOURG Cedex, FRANCE | Pr. Philippe Wolf (Formerly); Pr. Erik-André Sauleau (Currently) |
| 033031 | Eric Terrebonne | Comité de Protection des Personnes Est IV - 1, Place de l’Hôpital -67091 STRASBOURG Cedex, FRANCE | Pr. Philippe Wolf (Formerly); Pr. Erik-André Sauleau (Currently) |
| 033036 | David Tougeron | Comité de Protection des Personnes Est IV - 1, Place de l’Hôpital -67091 STRASBOURG Cedex, FRANCE | Pr. Philippe Wolf (Formerly); Pr. Erik-André Sauleau (Currently) |
| 033037 | Judith Raimbourg (Formerly: 20190424-20200923); Sandrine Hiret (Formerly: 20200924-20210214); Judith Raimbourg (Currently: Since 20210215) | Comité de Protection des Personnes Est IV - 1, Place de l’Hôpital -67091 STRASBOURG Cedex, FRANCE | Pr. Philippe Wolf (Formerly); Pr. Erik-André Sauleau (Currently) |
| 033038 | Christophe Borg | Comité de Protection des Personnes Est IV - 1, Place de l’Hôpital -67091 STRASBOURG Cedex, FRANCE | Pr. Philippe Wolf (Formerly); Pr. Erik-André Sauleau (Currently) |
| 033039 | Jean-Philippe Metges | Comité de Protection des Personnes Est IV - 1, Place de l’Hôpital -67091 STRASBOURG Cedex, FRANCE | Pr. Philippe Wolf (Formerly); Pr. Erik-André Sauleau (Currently) |
| 033040 | Louis-Marie Dourthe | Comité de Protection des Personnes Est IV - 1, Place de l’Hôpital -67091 STRASBOURG Cedex, FRANCE | Pr. Philippe Wolf (Formerly); Pr. Erik-André Sauleau (Currently) |
| 033042 | Farid El Hajbi | Comité de Protection des Personnes Est IV - 1, Place de l’Hôpital -67091 STRASBOURG Cedex, FRANCE | Pr. Philippe Wolf (Formerly); Pr. Erik-André Sauleau (Currently) |
| 033043 | Antoine Adenis | Comité de Protection des Personnes Est IV - 1, Place de l’Hôpital -67091 STRASBOURG Cedex, FRANCE | Pr. Philippe Wolf (Formerly); Pr. Erik-André Sauleau (Currently) |
| 033044 | François Ghiringhelli | Comité de Protection des Personnes Est IV - 1, Place de l’Hôpital -67091 STRASBOURG Cedex, FRANCE | Pr. Philippe Wolf (Formerly); Pr. Erik-André Sauleau (Currently) |
| 033048 | Pascal Artru | Comité de Protection des Personnes Est IV - 1, Place de l’Hôpital -67091 STRASBOURG Cedex, FRANCE | Pr. Philippe Wolf (Formerly); Pr. Erik-André Sauleau (Currently) |
| 049005 | Markus Moehler | Ethikkommission der Landesärztekammer Rheinland-Pfalz / Deutschhausplatz 3 55116 Mainz, GERMANY | Prof. Stephan Letzel |
| 049021 | Florian Lordick | Ethikkommission der Landesärztekammer Rheinland-Pfalz / Deutschhausplatz 3 55116 Mainz, GERMANY | Prof. Stephan Letzel |
| 049022 | Matthias Ebert | Ethikkommission der Landesärztekammer Rheinland-Pfalz / Deutschhausplatz 3 55116 Mainz, GERMANY | Prof. Stephan Letzel |
| 049024 | Gunnar Folprecht | Ethikkommission der Landesärztekammer Rheinland-Pfalz / Deutschhausplatz 3 55116 Mainz, GERMANY | Prof. Stephan Letzel |
| 049026 | Eray Goekkurt | Ethikkommission der Landesärztekammer Rheinland-Pfalz / Deutschhausplatz 3 55116 Mainz, GERMANY | Prof. Stephan Letzel |
| 049027 | Peter Thuss-Patience | Ethikkommission der Landesärztekammer Rheinland-Pfalz / Deutschhausplatz 3 55116 Mainz, GERMANY | Prof. Stephan Letzel |
| 049032 | Dirk Arnold | Ethikkommission der Landesärztekammer Rheinland-Pfalz / Deutschhausplatz 3 55116 Mainz, GERMANY | Prof. Stephan Letzel |
| 049033 | Thomas Zander | Ethikkommission der Landesärztekammer Rheinland-Pfalz / Deutschhausplatz 3 55116 Mainz, GERMANY | Prof. Stephan Letzel |
| 049035 | Frank Kullmann | Ethikkommission der Landesärztekammer Rheinland-Pfalz / Deutschhausplatz 3 55116 Mainz, GERMANY | Prof. Stephan Letzel |
| 039001 | Libero Ciuffreda | Comitato Etico Interaziendale AOU Città della Salute e della Scienza di Torino-AO Ordine Mauriziano-ASL Città di Torino / Corso Bramante, 88 Torino Torino 10126, ITALY | Marcello Maddalena |
| 039032 | Monica Lencioni | Comitato Etico Area Vasta Nord Ovest c/o Azienda Ospedaliero Universitaria Pisana / Via Roma 67, 56126 Pisa, ITALY | Romano Danesi |
| 039036 | Ferdinando De Vita | Comitato Etico Università degli Studi della Campania “Luigi Vanvitelli” - A.O.U. “Luigi Vanvitelli”- AORN “Ospedale dei Colli” / Via Costantinopoli, 104 80138, Napoli, ITALY | Prof. Liberato Berrino |
| 039037 | Giovanni Luca Frassineti | Comitato Etico Della Romagna CEROM / Via Piero Maroncelli, 40 Meldola Forli 47014, ITALY | Prof. Stefano Cascinu |
| 039038 | Luigi Cavanna | Comitato Etico dell’ Area Vasta Emilia Nord AVEN c/o Policlinico di Modena / Via Largo del Pozzo 71, 41124 Modena, ITALY | Sebastiano Calandra Buonaura |
| 039039 | Stefano Tamberi | Comitato Etico Della Romagna CEROM / Via Piero Maroncelli, 40 Meldola Forli 47014, ITALY | Prof. Stefano Cascinu |
| 039040 | Giorgio Vittorio Scagliotti | Comitato Etico Interaziendale A.O.U. San Luigi Gonzaga di Orbassano e AA.SS.LL. TO3 – TO4 – TO5 / Regione Gonzole,10 10043 Orbassano (TO), ITALY | Dr Mauro Felice Frascico (Formerly); Dr Antonio Giulio Piga (Currently) |
| 039044 | Roberto Bordonaro | Comitato Etico Catania 2 ARNAS Garibaldi Azienda Ospedaliera di Rilievo Nazionale e di Alta Specializzazione / P.zza S.M. di Gesù, 5, Catania, Catania, 95100, ITALY | Renato Bernardini |
| 034004 | Laura Visa Turmo (Formerly: 20181119-20200531); Carlos Gonzalez (Formerly, Temporary: 20200601-20210207); Laura Visa Turmo (Currently: Since 20210208) | Hospital Universitario Doce de Octubre, Comité de Etica de Investigación con Medicamentos, Hospital 12 de Octubre, Av. de Córdoba s/n 28041 Madrid, SPAIN | Dra. Mª del Puy Goyache Goñi |
| 034007 | Mariona Calvo Campos | Hospital Universitario Doce de Octubre, Comité de Etica de Investigación con Medicamentos, Hospital 12 de Octubre, Av. de Córdoba s/n 28041 Madrid, SPAIN | Dra. Mª del Puy Goyache Goñi |
| 034012 | Carlos Gómez Martin | Hospital Universitario Doce de Octubre, Comité de Etica de Investigación con Medicamentos, Hospital 12 de Octubre, Av. de Córdoba s/n 28041 Madrid, SPAIN | Dra. Mª del Puy Goyache Goñi |
| 034016 | Federico Longo Muñoz | Hospital Universitario Doce de Octubre, Comité de Etica de Investigación con Medicamentos, Hospital 12 de Octubre, Av. de Córdoba s/n 28041 Madrid, SPAIN | Dra. Mª del Puy Goyache Goñi |
| 034017 | Montserrat Blanco | Hospital Universitario Doce de Octubre, Comité de Etica de Investigación con Medicamentos, Hospital 12 de Octubre, Av. de Córdoba s/n 28041 Madrid, SPAIN | Dra. Mª del Puy Goyache Goñi |
| 034018 | Roberto Pazo | Hospital Universitario Doce de Octubre, Comité de Etica de Investigación con Medicamentos, Hospital 12 de Octubre, Av. de Córdoba s/n 28041 Madrid, SPAIN | Dra. Mª del Puy Goyache Goñi |
| 034020 | Miguel Marin Vera | Hospital Universitario Doce de Octubre, Comité de Etica de Investigación con Medicamentos, Hospital 12 de Octubre, Av. de Córdoba s/n 28041 Madrid, SPAIN | Dra. Mª del Puy Goyache Goñi |
| 034024 | Antonio Cubillo | Hospital Universitario Doce de Octubre, Comité de Etica de Investigación con Medicamentos, Hospital 12 de Octubre, Av. de Córdoba s/n 28041 Madrid, SPAIN | Dra. Mª del Puy Goyache Goñi |
| 034033 | Tamara Sauri | Hospital Universitario Doce de Octubre, Comité de Etica de Investigación con Medicamentos, Hospital 12 de Octubre, Av. de Córdoba s/n 28041 Madrid, SPAIN | Dra. Mª del Puy Goyache Goñi |
| 034034 | Maria Alsina Maqueda (Formerly: 20181122-20191028); Marc Diez (Currently: Since 20191029) | Hospital Universitario Doce de Octubre, Comité de Etica de Investigación con Medicamentos, Hospital 12 de Octubre, Av. de Córdoba s/n 28041 Madrid, SPAIN | Dra. Mª del Puy Goyache Goñi |
| 044010 | Hendrik-Tobias Arkenau | Health Research Authority / London Riverside REC, HRA, Skipton House, 80 London Road, London, SE1 6LH, UNITED KINGDOM | Dr Margaret Jones |
| 044012 | Richard Anthony Hubner | Health Research Authority / London Riverside REC, HRA, Skipton House, 80 London Road, London, SE1 6LH, UNITED KINGDOM | Dr Margaret Jones |
| 044015 | David Cunningham | Health Research Authority / London Riverside REC, HRA, Skipton House, 80 London Road, London, SE1 6LH, UNITED KINGDOM | Dr Margaret Jones |
| 044032 | Mark Harrison | Health Research Authority / London Riverside REC, HRA, Skipton House, 80 London Road, London, SE1 6LH, UNITED KINGDOM | Dr Margaret Jones |
| 044033 | Won-Ho Edward Park | Health Research Authority / London Riverside REC, HRA, Skipton House, 80 London Road, London, SE1 6LH, UNITED KINGDOM | Dr Margaret Jones |
| 044034 | Nicholas Robert Maisey | Health Research Authority / London Riverside REC, HRA, Skipton House, 80 London Road, London, SE1 6LH, UNITED KINGDOM | Dr Margaret Jones |
| 044037 | Anna Mary Young | Health Research Authority / London Riverside REC, HRA, Skipton House, 80 London Road, London, SE1 6LH, UNITED KINGDOM | Dr Margaret Jones |
| 044038 | David Cunningham | Health Research Authority / London Riverside REC, HRA, Skipton House, 80 London Road, London, SE1 6LH, UNITED KINGDOM | Dr Margaret Jones |
| 044043 | Mano Joseph | Health Research Authority / London Riverside REC, HRA, Skipton House, 80 London Road, London, SE1 6LH, UNITED KINGDOM | Dr Margaret Jones |
| 001003 | Igor Ivanovich Rybkin | Henry Ford Health Systems IRB / One Ford Place 2F, Detroit Michigan 48202, UNITED STATES | Jonathan Ehrman |
| 001080 | David J. Park | Advarra / 6940 Columbia Gateway Drive, Suite 110 Columbia, MD 21046, UNITED STATES | Sara Harnish |
| 001088 | Syma Iqbal | University of Southern California IRB / 1640 Marengo Street, Los Angeles California 90033, UNITED STATES | Micheal Bowdish |
| 001089 | Lindsey J. Graham (Formerly: 20180926-20190726); Jason K. Burris (Currently: 20190726-20200717) | Regional Health Command-Central IRB / 3551 Roger Brooke Drive, Fort Sam Houston, TX 78234-6315, UNITED STATES | Michael J Morris MD |
| 001096 | Dragana Tomic | Northwestern Memorial Healthcare, Institutional Review Board (NMHC IRB) / 25 North Winfield Road, Winfield Ilinois 60190, UNITED STATES | Gregory Kozeny |
| 001097 | Rex B. Mowat | Toledo Clinic Inc., IRB / 4235 Secor Rd., Toledo Ohio 43623, UNITED STATES | Curtis Black |
| 001098 | Anirudha Dasgupta | Advarra / 6940 Columbia Gateway Drive, Suite 110 Columbia, MD 21046, UNITED STATES | Sara Harnish |
